# Mechanical Thrombectomy for M1 Subocclusive Thrombus With Lateral Lenticulostriate Artery Occlusion: A Case Report and Literature Review

**DOI:** 10.3389/fneur.2022.828245

**Published:** 2022-02-07

**Authors:** Hirohisa Yajima, Satoshi Koizumi, Satoru Miyawaki, Nobuhito Saito

**Affiliations:** Department of Neurosurgery, The University of Tokyo Hospital, Tokyo, Japan

**Keywords:** subocclusion, mechanical thrombectomy, ischemic stroke, middle cerebral artery, lenticulostriate artery

## Abstract

The treatment for middle cerebral artery subocclusive thrombi is not standardized. Here, we report a case of M1 subocclusive thrombus with lateral lenticulostriate artery occlusion that was successfully treated with mechanical thrombectomy. This article describes a treatment strategy for M1 subocclusive thrombus, focusing on the indications for mechanical thrombectomy. A 58-year-old male on admission for pneumonia had a sudden onset of dysarthria and motor deficits. He has a history of dilated cardiomyopathy and underwent left ventricular assist device implantation 3 years ago. At onset, his National Institutes of Health Stroke Scale (NIHSS) score was nine. Computed tomography angiography demonstrated a filling defect in the distal right M1 segment of the middle cerebral artery. Angiography confirmed the presence of a subocclusive thrombus within the distal right M1 segment, although peripheral blood flow was maintained. Mechanical thrombectomy was performed for the M1 subocclusive thrombus using a direct aspiration first-pass technique, resulting in successful aspiration of the thrombus on the first pass. After the procedure, recanalization of the lateral lenticulostriate artery was detected, and the patient demonstrated full recovery (NIHSS score 0). Mechanical thrombectomy can be considered as a treatment option in cases of acute ischemic stroke caused by M1 subocclusive thrombus with lateral lenticulostriate artery occlusion, which presents with a high NIHSS score or neurological deterioration.

## Introduction

In contrast to complete occlusion, incomplete thrombotic vessel obstruction is called subocclusive thrombus or intraluminal non-occlusive free-floating thrombus (1). The internal carotid artery (ICA) is reportedly the most common location of subocclusive thrombus in the cervicocephalic arteries (2). However, literature describing middle cerebral artery (MCA) subocclusive thrombi is limited. While these thrombi can cause infarction, they are sometimes asymptomatic. Thus, the optimal treatment for subocclusive thrombi is still undefined. Here, we present a case of MCA subocclusive thrombus in the distal M1 segment with lateral lenticulostriate artery occlusion, for which mechanical thrombectomy was successful. Additionally, we propose a treatment strategy for suspected M1 subocclusive thrombus, with a literature review focusing on the indications for mechanical thrombectomy.

## Case Description

A 58-year-old male presented with a history of dilated cardiomyopathy. Three years ago, he had a left ventricular assist device (LVAD) implanted due to severe chronic heart failure. He had been taking warfarin to prevent thrombus formation in the LVAD circuit. He had a history of brain infarction, but with no neurological deficit and was listed for a heart transplant.

**Figure 1:**
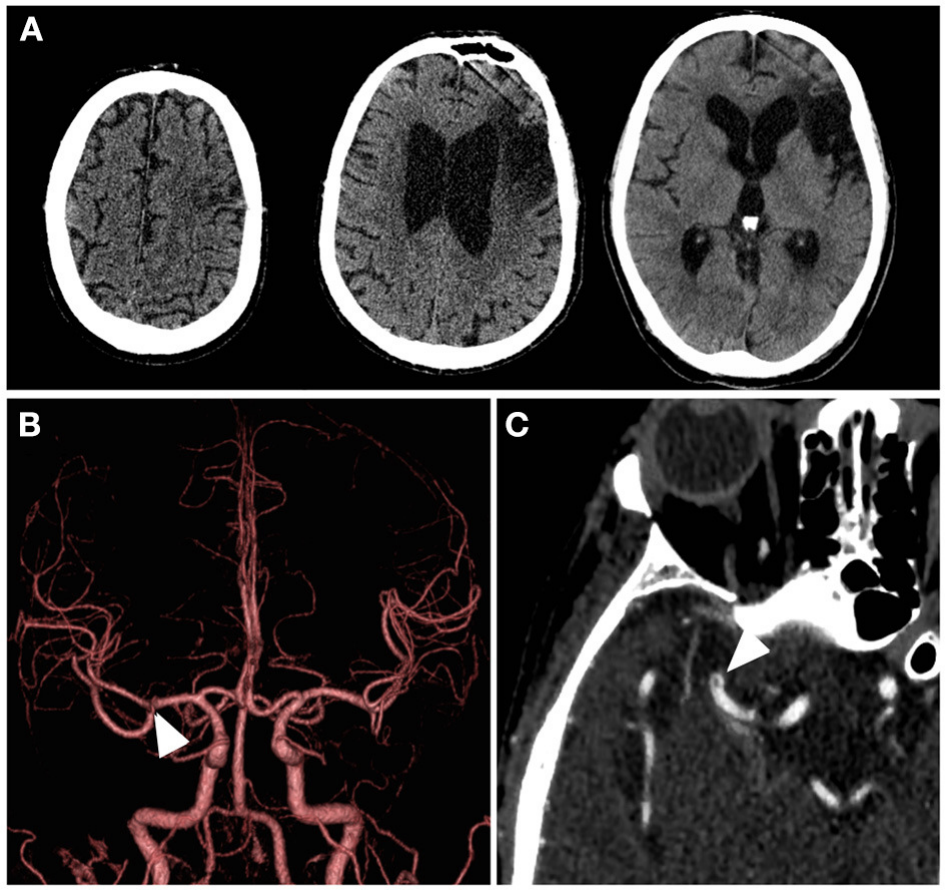
Patient's cerebral imaging at the time of symptom onset. **(A)** Computed tomography showed an obsolete infarction of the left frontal lobe, but there were no findings suggestive of hemorrhage or acute infarction on the diseased side. **(B)** Computed tomography angiography with three-dimensional volume rendering did not exhibit obvious filling defects, but a localized microstenosis was noted in the right middle cerebral artery distal M1 segment (white arrowhead). **(C)** Subocclusive thrombus in the right middle cerebral artery distal M1 segment (white arrowhead) was found on close observation through the axial image of the computed tomography angiography with maximum intensity projection.

The patient had a sudden-onset dysarthria and left hemiparesis while on admission for pneumonia. On neurological examination, his National Institutes of Health Stroke Scale (NIHSS) score was nine (facial palsy, one; left arm motor, four; left leg motor, three; dysarthria, one). Computed tomography (CT) showed an old cerebral infarction of the left frontal lobe, and no other new lesions that explain his symptoms had been noted (Figure 1A). Although CT angiography (CTA) did not demonstrate complete occlusion of the right MCA (Figure 1B), a localized microstenosis and a filling defect in the distal M1 segment of the MCA were identified on careful observation (Figures 1B,C).

**Figure 2:**
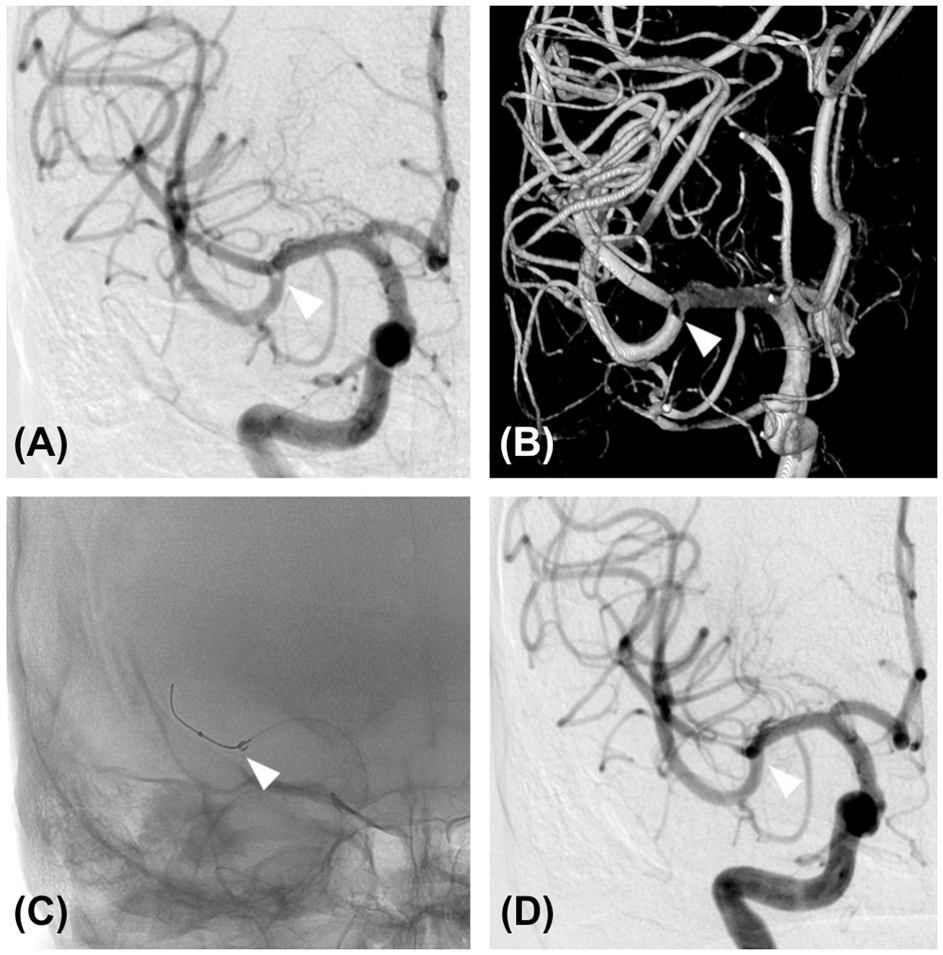
Diagnostic angiography and mechanical thrombectomy. **(A)** Cerebral angiography of the right internal carotid artery showing an M1 subocclusive thrombus (white arrowhead). **(B)** Three-dimensional reconstruction of the cerebral angiography of the right internal carotid artery also showed the presence of a thrombus (white arrowhead). **(C)** Mechanical thrombectomy was performed using a direct aspiration first-pass technique. An aspiration catheter was advanced to the location of the thrombus (white arrowhead) with the aid of a microcatheter and a micro guidewire. **(D)** Cerebral angiography of the right internal carotid artery after the first pass. Note the complete removal of the thrombus (white arrowhead).

The patient exhibited elevated prothrombin time international normalized ratio (PT-INR) at 3.31; therefore, he was not treated with either intravenous tissue plasminogen activator (tPA) or additional antithrombotic medication. For a definitive diagnosis, we decided to perform a cerebral angiography and mechanical thrombectomy. Right internal carotid angiography revealed the presence of a subocclusive thrombus within the distal M1 segment (Figures 2A,B). Considering that the chance for a heart transplant will become lower if the neurological deficit persists, mechanical thrombectomy for the subocclusive thrombus was performed.

**Figure 3:**
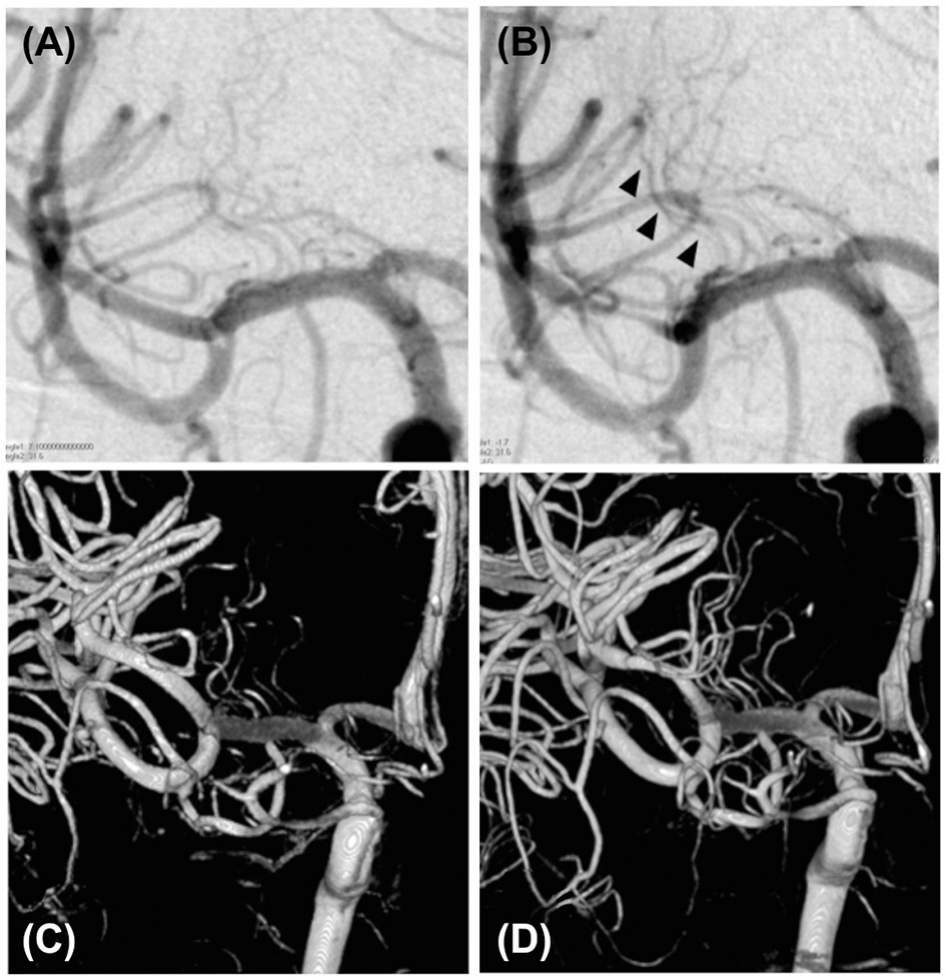
Visualization of the lateral lenticulostriate artery before and after the mechanical thrombectomy. **(A)** Cerebral angiography of the right internal carotid artery showing a suspected poor lateral lenticulostriate artery appearance before mechanical thrombectomy. **(B)** Cerebral angiography of the right internal carotid artery showing recanalization of the thrombus after mechanical thrombectomy (black arrowhead). **(C)** Three-dimensional reconstruction of the lateral lenticulostriate artery from the cerebral angiography of the right internal carotid artery, showing a poor appearance of the lateral lenticulostriate artery before the mechanical thrombectomy. **(D)** Three-dimensional reconstruction of the lateral lenticulostriate artery from the cerebral angiography of the right internal carotid artery showing improved lateral lenticulostriate artery appearance after mechanical thrombectomy.

The treatment was performed under local anesthesia without heparinization. Through femoral access with a 9-French long sheath, a 9 Fr Optimo balloon guiding catheter (Tokai Medical Products, Inc., Aichi, Japan) was placed into the ICA, and a Penumbra 5MAX ACE 60 reperfusion catheter (Penumbra, Alameda, CA, USA) was advanced into the thrombus within the distal right M1 segment over a coaxially inserted Phenom 27 microcatheter (Medtronic, Minneapolis, MN, USA) and CHIKAI black 14 soft tip micro guidewire (Asahi Intecc, Nagoya, Aichi, Japan). Mechanical thrombectomy was performed for subocclusive thrombus using a direct aspiration first-pass (ADAPT) technique (Figure 2C). Complete removal of the thrombus without distal migration (Figure 2D) and recanalization of the lateral lenticulostriate artery (LSA) was confirmed (Figures 3A–D), resulting in successful aspiration on the first pass. Immediately after the recanalization, there was complete recovery of his left hemiparesis. After mechanical thrombectomy, the narrowing of the M2 segment of the MCA was noted. Since the diameter of the blood vessels showed improvement over time compared to their original state, we concluded that the narrowing was spasm due to advancement of the tip of the aspiration catheter into the M2 superior trunk. The onset to picture time, picture to puncture time, and puncture to reperfusion time were 120, 60, and 40 min, respectively. Follow-up CT showed no hemorrhage or new ischemic lesion after the procedure. His postoperative NIHSS score was zero, and was discharged without any complications.

## Discussion

This report describes a case of M1 subocclusive thrombus with LSA occlusion that was successfully treated by mechanical thrombectomy. Most M1 subocclusive thrombi are treated conservatively. Moreover, through a literature search, we found only one case report of an M1 subocclusive thrombus (3). To date, there are no studies that have discussed the indications for mechanical thrombectomy in M1 subocclusive thrombus and its difficulty to diagnose.

Puetz et al. (4) reported that in 865 acute stroke and TIA cases evaluated by CTA, 10 cases (1.2%) had M1 subocclusive thrombi. This suggests that M1 subocclusive thrombus is not a rare condition. In previous reports (1, 3–5), the final diagnosis of M1 subocclusive thrombus was made using CTA or angiography. M1 subocclusive thrombi could be missed by magnetic resonance angiopgraphy (MRA) alone and may be diagnosed as lacunar infarction or branch atheromatous disease (BAD) (6). The neurological findings of lacunar infarction and BAD are reported to be relatively mild, with an NIHSS score of ≤ 7 and ≤ 5 for lacunar infarction (7) and BAD on admission, respectively. If the intracranial main artery has stenosis or occlusion, causing the symptom to be unclear on MRA despite a high NIHSS, CTA should be performed to investigate the subocclusive thrombi.

We reviewed the literature on conservative treatment for M1 subocclusive thrombi. To our knowledge, there are three reports describing 15 cases of M1 subocclusive thrombi with conservative treatment (1, 4, 5). Among these patients, nine (60%) presented with an NIHSS score of ≤ 4 points at the time of admission, and seven were discharged with an mRS score of ≤ 2. However, the two remaining patients exhibited neurological deterioration with poor prognosis (mRS of ≥3). In total, four patients (27%) had a poor prognosis. These findings suggest that conservative treatment usually leads to patient recovery. However, conservative management is insufficient when the presenting symptoms are severe or aggravated. In the case reported by Ohbuchi et al., medical management including intravenous tPA could not stop the neurological deterioration, hence mechanical thrombectomy was performed (3). There may be potential cases in which thrombectomy can be considered aggressively for patients with severe symptoms over refractory to conservative management.

An LSA recanalization was confirmed after the procedure in both the previous reports (3) and in our case. This suggests the effectiveness of LSA recanalization on M1 subocclusive thrombi, which may improve the functional prognosis of patients. Good LSA visualization after thrombectomy reportedly has a good functional prognosis for M1 complete occlusion (8). An LSA ischemia causes neurological deterioration (9), and the perfused area of the LSA has poor collateral circulation from other vessels, leading to early ischemia (10). Therefore, early reperfusion is desirable for LSA occlusion. In considering thrombectomy for M1 subocclusive thrombi, it may be useful to focus on the appearance of LSA.

Of note, our patient was on LVAD, a mechanical circulatory support device transplanted in patients with refractory heart failure awaiting heart transplantation (11). Although LVAD implantation improves prognosis and quality of life for a period of time (12), this device is a risk factor of hemorrhagic and ischemic stroke (13). For large vessel occlusion with patients on LVAD, mechanical thrombectomy can be performed safely and effectively (14, 15). We performed mechanical thrombectomy using ADAPT technique with a relatively low risk of perforation and postoperative hemorrhage (16). Due to the LVAD, MRI was contraindicated in this patient, which is a major limitation of this case report. It might be difficult to recognize small ischemic lesions postoperatively with only CT.

Therefore, mechanical thrombectomy can be considered for M1 subocclusive thrombi, especially in patients with a high NIHSS score or neurological deterioration resistant to conservative treatment. Additionally, LSA visualization may be an important factor in decision-making. Nevertheless, our study is limited by a lack of case series data. Further research is needed to determine an optimal treatment strategy for this condition.

## Conclusion

Based on our findings, mechanical thrombectomy can be a useful treatment option for selected cases of acute ischemic stroke caused by M1 subocclusive thrombus with LSA occlusion, which presents with a high NIHSS score or neurological deterioration.

## Data Availability Statement

The raw data supporting the conclusions of this article will be made available by the authors, without undue reservation.

## Ethics Statement

Ethical review and approval was not required for the study on human participants in accordance with the local legislation and institutional requirements. The patients/participants provided their written informed consent to participate in this study. Written informed consent was obtained from the individual(s) for the publication of any potentially identifiable images or data included in this article.

## Author Contributions

HY and SK: conceptualization, formal analysis, and investigation. HY: methodology, data curation, writing—original draft preparation, and visualization. SM, SK, and NS: validation, writing—review and editing, and supervision. SK: resources and project administration. All authors have read and agreed to the published version of the manuscript.

## Conflict of Interest

The authors declare that the research was conducted in the absence of any commercial or financial relationships that could be construed as a potential conflict of interest.

## Publisher's Note

All claims expressed in this article are solely those of the authors and do not necessarily represent those of their affiliated organizations, or those of the publisher, the editors and the reviewers. Any product that may be evaluated in this article, or claim that may be made by its manufacturer, is not guaranteed or endorsed by the publisher.
